# Neuroses Due to Anomalies of the Eye and Ear

**Published:** 1885-05

**Authors:** H. Gradle

**Affiliations:** 555 N. Clark Street; Chicago


### ARTICLE III.

### ON NERVOUS SYMPTOMS DUE TO OVERLOOKED ANOMALIES OF THE EYES AND EARS. *By* H. GRADLE, M. D., *of Chicago.*

[Read before the Chicago Medical Society, April 20th, 1885.]

In a paper read before the Illinois State Medical Association, at its last meeting, the author tried to point out the advantages of systematizing the search for the causes of disease,—not merely in theory, but also in the individual case. I there made a division of diseases as to their etiology, which though not new, indeed familiar to all of us, is sometimes not remembered at the right time. I distinguished between primary diseases and secondary affections. In the former instances some cause traceable to the outer world acts directly upon the parts involved in the disease, while, in the the latter class of cases, the symptoms are due to some other preëxisting primary disease, or lesion, or anomaly, somewhere in the body, perhaps at quite a distance. Of such secondary affections, I wish to present, to-night, various instances from my own observation, dependent upon anomalies of the eyes or ears. The special interest which these cases present is the fact that the local eye and ear symptoms were so insignificant that the primary trouble was either wholly overlooked—or at least not supposed to have any connection with the more annoying nervous symptoms in distant parts. The proof of this connection, however, was furnished in all these instances by the disappearance of the patient's complaints immediately following the proper treatment of the eye and ear difficulties. In none of these cases was any further active treatment pursued, except that coming within my special province.

The only region of the eye, the irritation of which I have ever known to cause disturbances at a distance, is the ciliary muscle. It is the condition of undue strain, to which this muscle is subjected, in hypermetropia and in astigmatism, which gives rise to so much remediable misery. The oculist sees almost daily people who have suffered for years from headaches and dizziness, and who are cured at once by a proper pair of glasses and perhaps the temporary use of atropine. But in most of these instances, the weakness of the eyes, the pain or the dimmed sight show the sufferer promptly that his eyes are at fault. It is hence not very often that we find the eye symptoms so slight as to escape the patient's attention. Still I have records of about twenty cases, observed during the last seven years, in which the patients, and generally their earlier medical attendants as well, had no definite idea that their misery was traceable to a defective state of their eyes.

The most frequent symptoms produced by eye-strain is headache. What I can say about this applies as well to the cases with manifest eye symptoms, as to those special instances in which no particular discomfort is experienced in the eyes. The headache is always frontal or temporal, though it may extend in nervous people over the entire head, even to the back of the neck. In some patients, the pain is constant; in another class, it occurs only after exertion of the eyes. But then such people usually know that the eyes are at fault. Perhaps most commonly a moderate pain exists, without intermission, but is made worse by work. The pain may be of variable severity. It is usually described as a dull ache. This headache, as well as the other symptoms to be described, has, in the experience upon which I base this paper, been permanently removed by the use of the proper spectacles. In some of the cases of astigmatism, it was necessary to keep the ciliary muscle paralyzed by the use of atropine or homatropine for a few days in order to have the glasses accepted.

It has been stated by several writers that hemicrania—the typical sick-headache—is sometimes due to refractive anomalies,—or rather to the eye-strain, which they engender. I cannot corroborate this from my own experience, tolerably large relative to refractive anomalies and at least fair in cases of migraine. I have never seen migraine cured by glasses alone. But I am ready to admit it may be one of the factors producing sick headache, and I have convinced myself that the correction of the eye-strain will facilitate the cure or at least the mitigation of migraine.

With or without headache, dizziness is a common result of eye-strain. This rarely amounts to any danger of falling or reeling, it is more commonly a dazed feeling, a difficulty of concentration upon any one subject, both of thought and of sight. Some patients have described it as a dullness scarcely distinguishable from a slight diffuse headache.

Not so common as the above complaints, have been symptoms of gastric disturbance in the patients under my notice. I have only seen four cases whose suspected stomach difficulty depended upon an overlooked anomaly of refraction, though I have had many other patients tell me that eye-strain, of which they were conscious, gave them a feeling of gastric uneasiness. The most remarkable one of these obscure stomach troubles was a man of thirty-three years, for whom I had prescribed moderately strong concave cylinders in spectacle frame, on account of intense eye-strain headaches and dizziness. Three years later I saw the patient again, well satisfied, as far as his eyes were concerned. He had worn the glasses with perfect satisfaction, but had had a new pair made six months previously. Since that time he felt somewhat dazed, and frequently slightly nauseated, had lost his appetite and had acquired a loathing for food. The glasses I found to be correct in strength, but they were fitted in the form of eye-glasses, and hence the direction of their axes was not quite constant. The patient had wondered himself whether the eye-glasses were to blame, inasmuch as there were no other signs present of actual intestinal disease, the tongue and the state of the bowels being normal, and no vomiting or abdominal pain having ever occurred. After the patient had returned for a few days to his glasses, set in a rigid frame, all symptoms of stomach disorder disappeared promptly.

Ear-lesions may cause disturbances at a distance, almost indifferently as to the part of the auditory organ in which they are situated. I have records of about fifteen cases, in which the aural treatment surprised the patients by curing their complaints in distant parts. Although disease of the ear does not produce nervous disturbances as often as eye-strain does, still I could refer to quite a large list of other patients who had distant symptoms, but had traced them themselves to the local disorder.

It is well known that irritation of the walls of the meatus will produce cough in many persons, ex. gr., on touching with instruments. The continued occurrence of this reflex was illustrated by the case of a young man who had had a cough for some four months, coming on sometimes in distressing fits, which several physicians had failed to remove, so that he had resolved to cease all treatment. An eczema of the meatus, which he supposed to have been there for several months, but to which he had not paid any attention, had lately spread further along the auricle, for which annoyance he called on me. After a single application of a strong solution of nitrate of silver to the well-denuded and scraped eczematous surface, his coughing fits never returned, and the slight bronchial irritation, which accompanied the trouble, disappeared during the next few days of treatment of the eczema. This cure of the cough was permanent, as I learned six weeks later. In another instance, a spasmodic cough in a child, the nocturnal attacks of which had kept the parents in a state of alarm for three weeks, was removed by taking a plug of exfoliated epithelial scales out of the auditory meatus.

In fact, this lesion—the scaling off of the epithelium of the meatus and its moulding into firm casts—I have found to be the most frequent ear disease causing distant symptoms. This peculiar disease of the epithelium is always accompanied by some interference with the permeability of the Eustachian tube, probably due to congestion, since it yields so readily to treatment, though less commonly it co-exists also with catarrh of the middle ear. The existence of these casts in the meatus does not always produce local symptoms, for the slight annoyance, to which they give rise, is overlooked by the less attentive patient, especially children. But in all my instances, the removal of the casts and the inflation of the middle ear made the ears and head feel clearer, even when the patient had considered his ears normal previously, perhaps on account of the gradual accustomance to the discomfort. The most frequent distant symptoms produced by this ear disease was headache, generally diffuse and often very persistent. In one case a young girl had been sent from the West to the East to consult some nerve-specialist, after having been treated by several prominent physicians without success. She had nothing but persistent dull headache, which made her disinclined to work or play, and hence, though never a sharp pain, proved a serious affliction. She was brought to my office to get my opinion whether possibly the eyes might be at fault, which, however, was not the case. I then examined the nose and found it normal, but saw some slight post-nasal catarrh. This led me to question her as to her ears, which she considered perfectly well, as, indeed, her hearing was practically normal. But there were the above-described casts in both ears and some obstruction of the Eustachian tube. The removal of these casts cleared her head considerably, and the inflation of the middle ear made her feel perfectly well immediately. She then admitted that the ears did feel better than before the consultation. The pain returned slightly some hours later, but was neither as annoying nor as persistent as it had been. It disappeared gradually during the next two weeks under treatment of the post-nasal catarrh and inflation of the middle ear. In two other instances this exfoliation of the skin of the meatus and the subsequent formation of solid plugs had caused irregular attacks of headache and a dull feeling for several months, until finally a severe irregularly intermittent neuralgia of the fifth nerve on the side of the affected ear led to the detection of the ear disease. While vertigo accompanies quite often any disease of the middle ear, which increases the intralabyrinthine pressure, I have never observed dizziness without the patient being aware of his ear trouble, except in very young children. I have at least once seen a child of 3 to 4 years of age reel and stagger during several days while suffering from an acute nasal-catarrh, which had involved the Eustachian tubes, but had not perceptibly injured the hearing. The immediate improvement following inflation of the middle ear dispelled the parents' anxiety about disease of the brain. In this connection, although perhaps foreign to the scope of this paper, I would call attention also to the frequency with which acute inflammation of the middle ear is overlooked in infants, whose intense suffering is ascribed to bowel disorder, sometimes even to pneumonia or brain-disease, until the discharge from the ear settles the diagnosis.

Spasmodic affections are occasioned not merely by irritatative lesions of the ear. There are on record several instances of epilepsy and of spasm of the ocular muscles—nystagmus—produced by the presence of foreign bodies in the meatus. I have not had any such instances under my own observation, but I have seen clonic spasms of the eye-lids, that is to say, continuous twitching and winking, in a child of six years who had slipped a bead into its left ear some three weeks previously. The bead had reddened the meatus slightly, but caused no further local irritation and the child had been afraid to mention the accident. Within two days after its removal the blepharospasm ceased. One instance of spasmodic wry-neck has come under my observation as the result of irritation of the meatus and the middle ear. A child four years of age had had an acute otitis of the right side. The treatment had either been not active enough or had been discontinued too early, for the otorrhœa had not ceased. But the discharge, being very slight, formed crusts which the mother took for wax, and as the child did not complain of pain and had fair hearing, the ear was considered cured. Some four months later spasm of the right sterno-cleido-mastoid muscle began, increasing for a week and then remaining about uniform during the child's waking hours. Medical treatment of different kinds during the next three weeks had no effect. A slight increase of the otorrhœa brought the child under my observation. I found a large polypus springing from the roof of the tympanum. After removing the same, the wry-neck became distinctly less, and disappeared entirely, together with the otorrhœa, on applying boracic acid powder during the following week.

555 N. Clark Street.

